# When is rotational angiography superior to conventional single‐plane angiography for planning coronary angioplasty?

**DOI:** 10.1002/ccd.26032

**Published:** 2015-05-27

**Authors:** Paul D. Morris, Jane Taylor, Sara Boutong, Sarah Brett, Amal Louis, James Heppenstall, Allison C. Morton, Julian P. Gunn

**Affiliations:** ^1^ Department of Cardiovascular ScienceUniversity of SheffieldUnited Kingdom; ^2^ Department of CardiologySheffield Teaching HospitalsSheffieldUnited Kingdom; ^3^ Insigneo Institute for In Silico MedicineSheffieldUnited Kingdom

**Keywords:** coronary angiography, rotational coronary angiography, percutaneous coronary intervention

## Abstract

**Objectives:**

To investigate the value of rotational coronary angiography (RoCA) in the context of percutaneous coronary intervention (PCI) planning.

**Background:**

As a diagnostic tool, RoCA is associated with decreased patient irradiation and contrast use compared with conventional coronary angiography (CA) and provides superior appreciation of three‐dimensional anatomy. However, its value in PCI remains unknown.

**Methods:**

We studied stable coronary artery disease assessment and PCI planning by interventional cardiologists. Patients underwent either RoCA or conventional CA pre‐PCI for planning. These were compared with the referral CA (all conventional) in terms of quantitative lesion assessment and operator confidence. An independent panel reanalyzed all parameters.

**Results:**

Six operators performed 127 procedures (60 RoCA, 60 conventional CA, and 7 crossed‐over) and assessed 212 lesions. RoCA was associated with a reduction in the number of lesions judged to involve a bifurcation (23 vs. 30 lesions, *P* < 0.05) and a reduction in the assessment of vessel caliber (2.8 vs. 3.0 mm, *P* < 0.05). RoCA improved confidence assessing lesion length (*P* = 0.01), percentage stenosis (*P* = 0.02), tortuosity (*P* < 0.04), and proximity to a bifurcation (*P* = 0.03), particularly in left coronary artery cases. X‐ray dose, contrast agent volume, and procedure duration were not significantly different.

**Conclusions:**

Compared with conventional CA, RoCA augments quantitative lesion assessment, enhances confidence in the assessment of coronary artery disease and the precise details of the proposed procedure, but does not affect X‐ray dose, contrast agent volume, or procedure duration. © 2015 Wiley Periodicals, Inc.

## INTRODUCTION

For 50 years, invasive coronary angiography (CA) has been the gold standard investigation for assessing coronary artery disease (CAD). CA remains the only investigation capable of selecting patients for and guiding coronary revascularization. CA techniques continue to improve in accessibility, resolution, radiation dose, image manipulation, and data storage.

CA is limited by consisting of a series of two‐dimensional “snapshots” of the coronary arteries, acquired from a limited number of “planes”. Appreciating the true three‐dimensional (3‐D) coronary and lesion anatomy requires the operator to recall the appearance of previously recorded angiographic runs and “reconstruct” the 3‐D anatomy using their imagination. This is subjective and unreliable 1, 2, 3. A number of studies based on intravascular ultrasound, angioscopy, and postmortem analysis have demonstrated how conventional, single‐plane CA may fail to adequately represent various anatomical characteristics, particularly in the context of complex CAD 4, 5, 6, 7, 8, 9.

Biplane angiography offers a partial solution by recording two orthogonal angiographic planes simultaneously. Although associated with reduced contrast use, the equipment required is not available in every center, the X‐ray dose is increased, and the number of views limited. Consequently, this technique has become more a feature of noncoronary, structural heart intervention 10.

**Figure 1:**
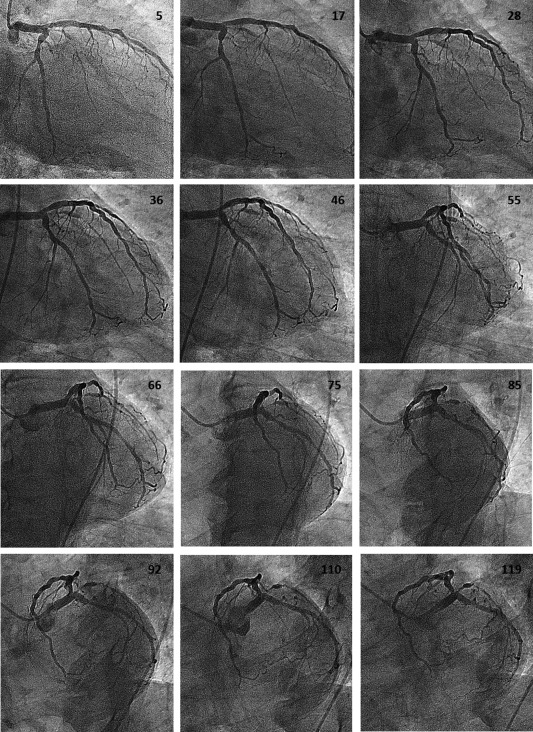
Representative frames from a typical rotational angiogram of a left coronary artery recorded in the caudal projection. The frame number is shown in each case.

**Figure 2:**
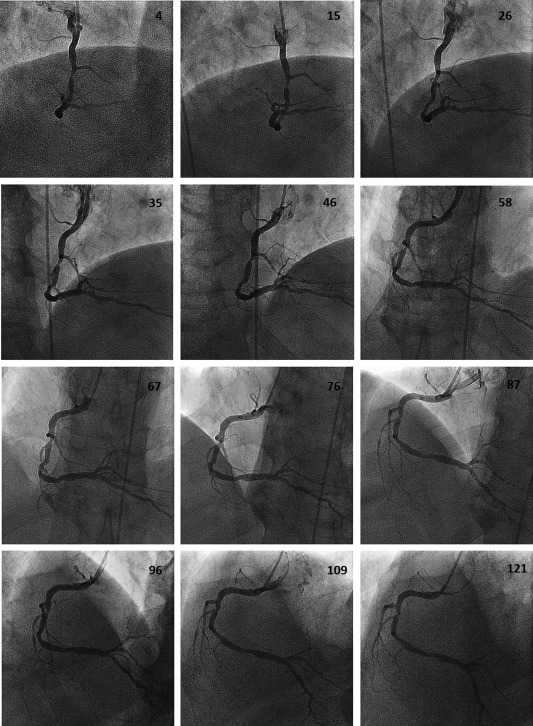
Representative frames from a typical rotational angiogram of a right coronary artery recorded in the cranial projection. The frame number is shown in each case.

Rotational CA (RoCA) is a relatively new method of angiographic image acquisition originally conceived and developed for imaging cerebral vessels, to overcome the limitations of conventional angiography 11, 12, 13. During RoCA, images are acquired as the X‐ray C‐arm rotates around the patient, in a transverse axis (typically an arc of 120°, at 30° per second), recording 121 sequential two‐dimensional images, with or without cranial or caudal tilt (Figs. 1 and 2) 14. RoCA has several advantages over traditional CA. First, in the context of diagnostic CA, RoCA is associated with a reduction in the volume of contrast agent used and the total radiation dose 14, 15, 16, 17. RoCA has, therefore, gained popularity in patients with renal insufficiency 18. Second, RoCA provides 121 separate views and, therefore, may reveal more anatomical detail than conventional CA. Third, RoCA is viewed as a single run, whereas conventional CA requires the operator or radiographer to “scroll” between multiple single‐plane acquisitions 19. Fourth, using image segmentation software, RoCA image data can be used to generate a 3‐D reconstruction of vessel geometry, which can be manipulated on a desktop computer to aid planning of percutaneous coronary intervention (PCI) 20, a technique that can be used to generate 3‐D geometric models for computing intracoronary physiological parameters such as fractional flow reserve 21.

Immediately prior to PCI, a “planning” CA was performed, which assesses any changes since the diagnostic CA, any unclear aspects of the coronary circulation or lesion, and to determine the optimal strategy for PCI. RoCA has become established in the context of diagnostic CA, but its role in pre‐PCI planning is yet to be established. The aim of this study was to compare RoCA and conventional CA in the context of pre‐PCI strategy planning in terms of lesion assessment, operator confidence, procedure time, volume of contrast agent infused, and radiation dose delivered.

## MATERIALS AND METHODS

### Location and Design

This was an observational study performed at the South Yorkshire Cardiothoracic Centre, Sheffield Teaching Hospitals NHS Foundation Trust. The study complied with local research ethics committee guidance.

### Population

Patients with stable, native vessel CAD referred for PCI were studied. Patients with graft lesions, chronic total occlusion, acute presentation, or severe truncal obesity were excluded.

### Clinical Protocol

**Figure 3:**
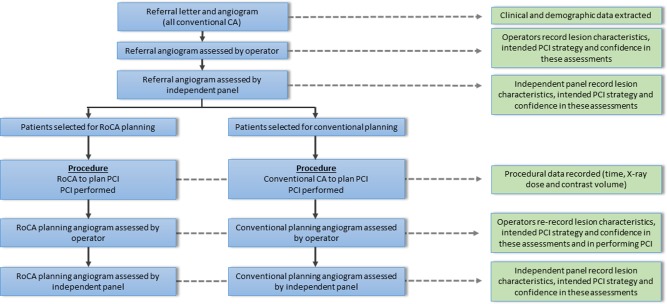
Flowchart of the study protocol (blue) and data collection (green).

Patients were selected for standard CA or RoCA planning according to operator preference (nonrandom), maintaining a balance between both techniques. Operators were accustomed to both techniques. Baseline clinical data were extracted from hospital records. Referral CAs (all conventional) were evaluated, by the operator, who recorded details of the lesion, the proposed PCI strategy, and their level of confidence (0–10, with 10 indicating highest confidence) regarding each element of angiographic assessment and PCI planning. Operators re‐evaluated these assessments, on the basis of the planning angiogram (RoCA or standard CA) before proceeding to PCI according to standard practice. X‐ray dosage, contrast usage, and procedure time were recorded. Operators also graded their level of confidence associated with each angiographic method in terms of lesion assessment and PCI planning. Planning CAs were then compared with referral CAs, to evaluate any added value associated with the different techniques in terms of planning PCI. All parameters were re‐evaluated by an independent panel. Figure 3 outlines the study protocol. Details of the patient and PCI evaluation record are included in the Supporting Information Appendix.

### Angiographic Protocols

Conventional multiple, single‐plane CA was performed according to standard practice. Individual projection angles were selected at the operators' discretion. RoCA was performed (Allura 3D‐RA, Philips Healthcare, Best, NL), after iso‐centering in posterior–anterior and lateral planes on a breath hold, with a single hand injection of 15–20 mL contrast. Right coronary artery (RCA) target vessels underwent a single RoCA with 25° cranial tilt, and left coronary artery (LCA) cases underwent two RoCAs, one with 25° cranial tilt and one with 25° caudal tilt, to ensure comprehensive assessment of all lesions and branches.

### Statistics

Continuous data are expressed as mean (standard deviation) and were compared using paired or unpaired Student's *t*‐tests, as appropriate. Categorical data are expressed as number and/or percentage and were compared using Pearson's *χ* ^2^ test or the Wilcoxon sign‐rank test. Between‐group differences in confidence scores were compared using the Mann–Whitney *U*‐test. Fisher's exact test was used to analyze 2 × 2 tables. Statistical significance was considered at the 5% level.

## RESULTS

Six PCI operators performed 127 procedures over a 7‐month period. Sixty‐six patients underwent RoCA and 61 conventional CA as their planning angiogram. All six operators contributed cases to both groups, with a balance between conventional and rotational techniques (J.P.G., 25 and 27; S.B., 19 and 16; A.L., 8 and 7, T.R., 5 and 7; and others, 4 and 9). Seven patients (5.5%; six RoCA and one conventional CA) crossed‐over to the other modality, which could be regarded as “failure” of the initial strategy as a complete assessment tool (*P* = 0.11). These seven were excluded from subsequent analyses. The mean patient age was 64 years, weight was 80 kg, height was 1.70 m, and 62% were men. The mean number of significant lesions was 1.8, vessels treated was 1.5, balloons used was 2.8, and stents deployed was 2.0. There were no significant differences between the two groups in terms of baseline demographics, medical history, or type of PCI procedure performed (Table 1). In total, 212 significant lesions were assessed for potential treatment (97 conventional and 115 RoCA; 61 RCA and 151 LCA).

**Table 1:** Patient and Procedure Characteristics of the Groups Undergoing RoCA and Conventional CA for Planning PCI

	Planning angiogram		
	Conventional CA (*n* = 60)	RoCA (*n* = 60)	*P*
Patient characteristics			
Age (years)	64.7 (10.8)	63.5 (13.2)	0.59
Male sex	34 (56.7%)	40 (66.7%)	0.26
Weight (kg)	80.2 (14.3)	81.1 (17.1)	0.70
Height (m)	1.7 (0.1)	1.7 (0.1)	1.0
PCI data			
Vessels treated	1.4 (0.6)	1.6 (0.8)	0.12
Lesions treated	1.6 (0.9)	1.9 (1.3)	0.14
Balloons used	2.7 (2.3)	3.0 (2.5)	0.50
Stents used	1.8 (1.3)	2.2 (1.6)	0.14

Data presented as mean (SD) except for Male Sex.

CA, coronary angiography; PCI, percutaneous coronary intervention; RoCA, rotational coronary angiography.

### Lesion Assessment and PCI Strategy

Some lesions that had previously been judged by the operators on the basis of the referral CA as nonsignificant were deemed, during the planning CA, to be significant, and vice versa. However, there was no significant difference in the frequency of lesions being excluded or additional lesions included between the RoCA and conventional groups (see Supporting Information Appendix Table A1).

### Quantitative Lesion Assessment

Paired comparison analysis of lesion characteristics (Table 2) revealed that using RoCA to plan PCI resulted in a significantly different assessment of vessel caliber and lesion involvement with a bifurcation, compared with the referral CA. Operators and the independent panel found that, compared with the conventional referral CA, using RoCA led to a reduction in the assessment of vessel caliber (2.8 mm vs. 3.0 mm, *P* < 0.05) and a reduction in the number of lesions deemed to involve a bifurcation (23 vs. 30 lesions, *P* < 0.05). Using conventional CA to plan PCI did not alter lesion characteristic assessment.

**Table 2:** Assessment of Lesion Characteristics by the Independent Panel's Examination of the Referral and Planning CAs (Conventional CA or RoCA)

	Conventional CA			RoCA		
	Referral CA	Planning CA	*P*	Referral CA	Planning CA	*P*
Stent length (mm)	15.4 (8.1)	15.5 (8.2)	0.78	17.1 (9.0)	17.9 (10.0)	0.08
Vessel size (mm)	3.1 (0.6)	3.1 (0.6)	0.87	3.0 (0.6)	2.8 (0.6)	0.02
Stenosis (%)	74.8 (10.5)	73.6 (15.2)	0.21	72.7 (9.6)	73.7 (14.1)	0.33
Irregular (*n*)	40 (41)	41 (42)	0.80	55 (48)	60 (52)	0.17
Eccentric (*n*)	24 (25)	21 (22)	0.49	37 (32)	29 (25)	0.07
Tortuous (*n*)	11 (11)	11 (11)	1.0	10 (9)	12 (10)	0.16
Ostial (*n*)	17 (18)	17 (18)	1.0	16 (14)	13 (11)	0.08
Bifurcation (*n*)	19 (20)	19 (20)	1.0	30 (26)	23 (20)	0.03
Calcification (*n*)	25 (26)	30 (31)	0.17	35 (30)	43 (37)	0.07
Angulation (degrees)	3 (3.6)	5 (6)	0.16	8 (7.6)	9 (8.6)	0.66
Thrombus (*n*)	2 (2.4)	2 (2.4)	1.0	0 (0)	0 (0)	1.0

Data presented as mean (SD) or *n* (%).

CA, coronary angiography; RoCA, rotational coronary angiography.

### Confidence Levels

Overall operator confidence was significantly greater with RoCA planning compared with conventional CA (confidence level on a scale of 0–10 was 8.9 vs. 8.2, respectively; *P* < 0.05). In terms of the perceived number of significant lesions, both the operators and the independent expert panel were more confident following RoCA compared with conventional CA (increase in confidence level 0.9 vs. 0.5 for the operators and 0.6 vs. 0.3 for the panel; *P* < 0.05 for both). In the assessment of certain lesion characteristics (lesion length, % stenosis, tortuosity, and angulation), there was also a greater increase in confidence among operators and the panel with RoCA compared with conventional CA (Table 3).

**Table 3:** Increase in Confidence Level (Scale 0–10) in the Assessment of Selected PCI Parameters From Referral CA (Conventional in All Cases) to Planning CA (RoCA or Conventional), Operator and Panel Analysis

	Operators			Independent panel		
Lesion characteristic	Conventional (*n* = 97 lesions)	RoCA (*n* = 115 lesions)	*P*	Conventional (*n* = 97 lesions)	RoCA (*n* = 115 lesions)	*P*
Length	0.4 (1.0)	1.0 (1.1)	<0.01	0.3 (0.9)	0.7 (1.0)	0.01
Caliber	0.7 (0.8)	0.8 (0.9)	0.19	0.4 (0.7)	0.5 (0.8)	0.48
% Stenosis	0.4 (0.9)	0.9 (0.9)	<0.01	0.4 (1.0)	0.7 (0.8)	0.02
Irregularity	0.3 (0.8)	0.6 (0.8)	0.01	0.2 (0.5)	0.4 (0.8)	0.11
Eccentricity	0.3 (0.8)	0.7 (0.7)	<0.01	0.3 (0.6)	0.5 (0.8)	0.31
Tortuosity	0.3 (0.5)	0.4 (0.6)	0.02	0.1 (0.3)	0.2 (0.4)	0.04
Ostial	0.0 (0.2)	0.1 (0.5)	0.17	0.0 (0.4)	0.0 (0.4)	0.88
Bifurcation	0.3 (0.6)	0.5 (0.8)	0.08	0.2 (0.5)	0.3 (0.7)	0.21
Calcification	0.4 (0.7)	0.4 (0.8)	0.84	0.3 (0.8)	0.2 (1.0)	0.72
Angulation	0.3 (0.6)	0.5 (0.7)	0.06	0.1 (0.3)	0.3 (0.5)	<0.01
Thrombus	0.2 (0.5)	0.2 (0.6)	0.56	0.1 (0.5)	0.2 (0.4)	0.05
Strategy	0.5 (0.9)	0.7 (0.8)	0.12	0.2 (0.7)	0.4 (0.7)	0.12
View	0.6 (0.9)	0.7 (1.0)	0.33	0.4 (0.9)	0.6 (1.1)	0.60
Predilatation	0.2 (0.6)	0.2 (0.5)	0.68	0.1 (0.4)	0.2 (0.6)	0.06
Size of stent	0.6 (0.9)	0.8 (0.9)	0.25	0.4 (0.6)	0.4 (0.6)	0.54
Type of stent	0.4 (0.8)	0.5 (0.9)	0.45	0.3 (0.7)	0.3 (0.7)	0.76

Data presented as mean (SD).

CA, coronary angiography; RoCA, rotational coronary angiography.

### Left Vs. Right Coronary Artery

Among operators and the panel, there was a greater increase in confidence level in assessing lesion characteristics (both RoCA and conventional CA) for LCA cases, compared with the RCA cases. However, there was added value in terms of increased confidence in assessing several lesion characteristics (length, % stenosis, irregularity, and angulation) with RoCA rather than conventional CA in the LCA cases (Supporting Information Appendix Table A1). This was not the case in RCA cases (Supporting Information Appendix Table A3).

### Procedure Time, Radiation Dose, and Contrast Volume

There were no statistically significant differences in total procedure time, X‐ray dose, screening time, cine runs, or contrast usage between PCIs guided by the two techniques, although all trends favored conventional CA over RoCA (Table 4).

**Table 4:** Procedure Duration, Screening Time, X‐ray Dose and Volume of Contrast Used

	Conventional CA, mean (SD)	RoCA, mean (SD)	*P*
*n*	60	60	
Procedure duration (min)	58.2 (34.2)	67.2 (36.9)	0.17
Screening time (min)	14.5 (10.8)	14.1 (11.5)	0.84
X‐ray dose (Gy cm^2^)	47.5 (35.1)	59.7 (40.1)	0.08
Total number runs	25.6 (15.9)	26.1 (17.0)	0.86
Pre‐PCI runs	4.3 (2.1)	3.7 (2.0)	0.11
Contrast volume (mL)	310.9 (186.5)	342.4 (188.1)	0.36

Data presented as mean (SD).

CA, coronary angiography; RoCA, rotational coronary angiography; PCI, percutaneous coronary intervention.

### Supplemental Diagnostic Runs

Thirty‐one of 60 (52%) planning RoCAs were supplemented with an additional single‐plane (conventional) acquisition. Most commonly (22 cases), this was a supplementary left anterior oblique caudal (“spider”) view to better assess the distal left main stem and proximal left anterior descending artery and circumflex. In two of 60 (3%) conventional CA cases, RoCA was added.

## DISCUSSION

This is the first study to investigate the use of RoCA in the context of planning PCI; comparing RoCA against conventional single‐plane CA for immediate pre‐PCI planning. RoCA improved the operator's understanding of the target lesions and the proposed procedure compared with the knowledge gained from the diagnostic (conventional) CA. RoCA resulted in a reduction in the number of lesions judged to involve a bifurcation and a reduction in the assessed vessel caliber. These parameters directly influence PCI strategy, particularly in terms of stent sizing and deployment. RoCA may, therefore, provide additional information in terms of quantitative lesion measurements and proposed stent parameters. Whether or not this corresponds with a reduction in complications and negative outcomes (arising from stent over‐sizing and procedures that unnecessarily involve a bifurcation) remains to be determined.

A further advantage of RoCA was in the level of confidence demonstrated by the operator in terms of understanding of the 3‐D anatomy and the lesion characteristics, particularly in the LCA. The parameters in which this was noted were lesion length, percentage stenosis, lesion irregularity, and the degree of tortuosity. Unlike in diagnostic CA, planning RoCA inferred no advantages in terms of reducing X‐ray dose, contrast usage, nor procedure or screening time.

Any advantage conferred by a greater confidence level when performing PCI is unclear, and this study did not aim to address that. It may be speculated that improved confidence might translate into better patient safety or even long‐term results, although it would require much larger large study to prove that. There might also be an economic advantage if the correct stent type and length is selected, or by deploying a more trackable device in a tortuous vessel. A particular advantage of RoCA vs. conventional CA was observed in assessment of the LCA rather than the RCA, presumably because of greater 3‐D complexity manifested in vessel overlap and branching in the LCA, which is not the case in the RCA. No advantage was found when RoCA, rather than conventional CA, was used in the RCA.

RoCA has been installed in many cardiac catheter laboratories, and is a well‐established method of imaging the coronary arteries 16, 22. Yet it is rarely used, either in the diagnostic role or in the role of PCI planning. The reasons are well known to operators who use the technique, and may be inferred from the trend observed in our study toward an increased procedure time with RoCA, which occurs because of the requirement for careful iso‐centering and a “dummy run” and the frequent proximity warnings (activated if the C‐arm approaches the patient), which necessitate adjustments to drapes, patient position, and table height before re‐starting the whole process. In addition, errors in iso‐centering (cutting off a vessel), insufficient contrast in the syringe, or disengagement of the catheter requires RoCA to be repeated. Even good‐quality RoCAs may have to be supplemented with conventional runs (52% in this study), usually because the degree of cranial or caudal angulation is insufficient to visualize the proximal LCA. Finally, RoCA is impossible in very obese patients.

There was no advantage in terms of radiation dose, contrast usage, or procedure time with RoCA compared with conventional CA in the pre‐PCI planning role, despite reductions in these parameters being documented in the role of diagnostic CA in several studies 14, 15, 16, 17. The reasons for this are twofold. First, regarding contrast and X‐ray dose, the pre‐PCI set‐up CA is only one small part of the procedure; conventional single‐plane CAs are used to guide the procedure as it progresses in both groups. Therefore, any small savings in contrast and radiation at the first stage will be diluted by the expenditure of each during the course of the procedure. Second, regarding time, accurate setting up of a good‐quality RoCA takes longer than acquiring three or four single‐plane conventional CAs.

### Limitations of This Study

This was an observational, rather than a randomized study. However, operators were competent at both conventional CA and RoCA and selected the modality themselves in approximately equal numbers without significant bias. An attempt to limit any potential bias was made by incorporating a parallel analysis by a panel, offline, and also the baseline characteristics of the patients, vessels, and lesions in each group were remarkably similar. This was a single‐center study, although this conferred the advantage of consistency of methodology. The numbers included were modest. Outcome measures related to operator assessments rather than patient outcomes.

## CONCLUSIONS

In the context of planning PCI, RoCA may offer advantages of better appreciation of lesions and planning of the procedure than conventional CA, particularly in LCA cases. In contrast to purely diagnostic RoCA, it does not reduce the volume of contrast used, the X‐ray exposure, or the procedure time. It has several practical disadvantages in a busy interventional catheter laboratory. It is not known whether these procedural advantages translate into clinical benefit for patients or training benefit for interventional cardiologists.

## Supporting information

Supporting Information

Supporting Information

Supporting Information
