# Supplementary material for: When is rotational angiography superior to conventional single‐plane angiography for planning coronary angioplasty?
Source: Catheter Cardiovasc Interv. 2015 May 27;87(4):E104–12. doi: 10.1002/ccd.26032 (PMC4855622; doi:10.1002/ccd.26032)
Supplement: Supplementary file 1 — Supporting Information [file CCD-87-E104-s001.docx]

***E-appendix: supplementary data for materials and methods***

***Patient /percutaneous coronary intervention evaluation record***

Baseline clinical and demographic data extracted from the patient record included: age (date of birth), gender, NHS number, weight, height and body mass index (BMI). Procedural data included: the identity of the primary operator, total procedure time (min), X-ray screening time (min), X-ray dose (Gycm2) and contrast volume (ml). Recorded PCI data included: vessel treated, lesion treated, balloons used, stents used and the number of pre-procedural and procedural angiographic acquisitions (CA, RoCA, LCA or RCA).

Diagnostic CAs (all conventional CA) were studied by the operator. Based upon this assessment, their intended procedure was recorded. Recorded details included the vessel/s they intended treating, the number of lesions judged to require revascularization, along with the length (mm) and caliber (mm) of stent they would ideally select for each lesion. At this point, operators also recorded lesion assessment details. Lesion assessment was based upon the American College of Cardiology (ACC) and American Heart Association (AHA) lesion morphology classification system. Operators logged lesion length (mm), vessel caliber (mm), percentage lumen stenosis (%), irregularity (Y/N), eccentricity (Y/N), tortuosity (Y/N), involvement of the ostium (Y/N), involvement of a bifurcation (Y/N), calcification (Y/N), angulation (degrees), presence of thrombus (Y/N) and length of stent selected (mm). Only lesions which were deemed to warrant intervention were scored. Operators recorded the same details after the planning angiogram (RoCA or conventional) to assess differences. During the procedure, the number, and type of balloons and stents deployed and for which lesion, were recorded.

Operator confidence was scored out of 10 (10 indicating highest confidence) in terms of their ability to interpret vessel and lesion characteristics and planning PCI strategy (as above). Confidence was assessed pre-procedure (on the basis of the diagnostic /referral CA) and again after the planning CA performed immediately prior to performing PCI. Operators scored their confidence in judging: quality of view, whether to pre-dilate, stent choice (length and width), extent of disease, number of significant lesions, and lesion characteristics (see above).

After PCI, operators noted any supplementary acquisitions they had recorded additional to the selected technique i.e. if they had selected RoCA for PCI planning, were any conventional CA runs acquired and if so why?

All of the above analyses were repeated, offline by a blinded independent panel.

**Table A1.** *Number of significant lesions added or discounted for PCI by the planning angiogram (conventional CA vs RoCA)*

| Significant lesions | Conventional CA | RoCA | *P* |
| --- | --- | --- | --- |
| n lesions | 97 | 115 |  |
| Added after planning CA | 3 (3.1) | 6 (5.2) | 0.355 |
| Discounted after planning CA | 6 (6.2) | 9 (7.8) | 0.445 |
| Added during PCI | 6 (6.2) | 4 (3.5) | 0.513 |
| Discounted during PCI | 7 (7.2) | 5 (4.3) | 0.580 |

CA; coronary angiography, PCI; percutaneous coronary intervention, RoCA; rotational coronary angiography. Data presented as n (%).

**Table A2.** *Increase in confidence level (scale 0-10) between diagnostic and planning angiogram (conventional CA or RoCA) for LCA vessels, operator and panel analysis.*

|  | Operator | | | Independent panel | | |
| --- | --- | --- | --- | --- | --- | --- |
| Characteristic | Conventional  (n=58) | RoCA  (n=93) | *P* | Conventional  (n=58) | RoCA  (n=93) | *P* |
| Length | 0.5 (1.0) | 1.1 (1.0) | <0.01 | 0.3 (0.9) | 0.8 (1.0) | 0.01 |
| Caliber | 0.8 (0.8) | 0.9 (0.9) | 0.46 | 0.5 (0.7) | 0.6 (0.8) | 0.34 |
| Stenosis | 0.5 (0.9) | 0.9 (0.9) | 0.03 | 0.5 (1.0) | 0.8 (0.9) | 0.05 |
| Irregularity | 0.4 (0.9) | 0.6 (0.8) | 0.04 | 0.3 (0.5) | 0.5 (0.8) | 0.04 |
| Eccentricity | 0.4 (0.9) | 0.7 (0.8) | 0.01 | 0.3 (0.7) | 0.5 (0.8) | 0.24 |
| Tortuosity | 0.3 (0.6) | 0.5 (0.6) | 0.13 | 0.1 (0.3) | 0.3 (0.4) | 0.06 |
| Ostial | 0.0 (0.3) | 0.1 (0.6) | 0.24 | 0.0 (0.5) | 0.1 (0.5) | 0.94 |
| Bifurcation | 0.3 (0.7) | 0.6 (0.8) | 0.08 | 0.2 (0.6) | 0.4 (0.8) | 0.17 |
| Calcification | 0.5 (0.7) | 0.3 (0.8) | 0.14 | 0.3 (0.7) | 0.2 (0.7) | 0.37 |
| Angulation | 0.3 (0.6) | 0.5 (0.7) | 0.05 | 0.1 (0.3) | 0.3 (0.5) | 0.02 |
| Thrombus | 0.2 (0.5) | 0.2 (0.6) | 0.65 | 0.1 (0.6) | 0.2 (0.4) | 0.10 |
| Strategy | 0.5 (0.9) | 0.7 (0.8) | 0.17 | 0.2 (0.8) | 0.5 (0.8) | 0.13 |
| View | 0.7 (1.1) | 0.8 (1.1) | 0.79 | 0.7 (0.8) | 0.7 (1.2) | 0.97 |
| Pre-dilate | 0.3 (0.7) | 0.1 (0.4) | 0.30 | 0.2 (0.5) | 0.3 (0.6) | 0.20 |
| Size of stent | 0.6 (1.0) | 0.7 (0.9) | 0.73 | 0.4 (0.6) | 0.5 (0.6) | 0.13 |

CA; coronary angiography, RoCA; rotational coronary angiography. Data presented as mean (SD).

**Table A3.** *Increase in confidence level (scale 0-10) between referral and planning angiogram (conventional CA or RoCA) for RCA vessels, operator and panel analysis.*

|  | Operator | | | Independent panel | | |
| --- | --- | --- | --- | --- | --- | --- |
| Characteristic | Conventional  (n=39) | RoCA  (n=22) | *P* | Conventional  (n=39) | RoCA  (n=22) | *P* |
| Length | 0.4 (1.1) | 0.8 (1.1) | *0.19* | 0.3 (1.0) | 0.7 (0.9) | *0.20* |
| Caliber | 0.4 (0.7) | 0.6 (0.7) | *0.26* | 0.4 (0.7) | 0.4 (0.7) | *0.78* |
| Stenosis | 0.2 (0.8) | 0.7 (0.8) | *0.02* | 0.3 (0.9) | 0.5 (0.5) | *0.20* |
| Irregularity | 0.2 (0.6) | 0.6 (0.7) | *0.07* | 0.1 (0.6) | 0.1 (0.6) | *0.57* |
| Eccentricity | 0.1 (0.4) | 0.6 (0.7) | *0.01* | 0.3 (0.6) | 0.3 (0.6) | *0.83* |
| Tortuosity | 0.1 (0.4) | 0.4 (0.7) | *0.03* | 0.1 (0.2) | 0.1 (0.4) | *0.62* |
| Ostial | 0.0 (0.0) | 0.0 (0.4) | *0.55* | 0.1 (0.2) | 0.0 (0.3) | *0.57* |
| Bifurcation | 0.2 (0.5) | 0.2 (0.6) | *0.91* | 0.1 (0.3) | 0.1 (0.2) | *0.55* |
| Calcification | 0.0 (0.4) | 0.4 (0.8) | *0.03* | 0.4 (1.0) | 0.4 (1.4) | *0.52* |
| Angulation | 0.3 (0.6) | 0.3 (0.6) | *0.87* | 0.0 (0.3) | 0.2 (0.4) | *0.10* |
| Thrombus | 0.2 (0.5) | 0.3 (0.5) | *0.68* | 0.1 (0.4) | 0.2 (0.5) | *0.29* |
| Strategy | 0.5 (1.0) | 0.6 (0.8) | *0.55* | 0.1 (0.4) | 0.1 (0.2) | *0.97* |
| View | 0.3 (0.6) | 0.6 (0.9) | *0.22* | -0.1 (0.6) | 0.2 (0.8) | *0.38* |
| Pre-dilate | 0.1 (0.4) | 0.2 (0.5) | *0.38* | -0.1 (0.2) | 0.1 (0.3) | *0.09* |
| Size of stent | 0.4 (0.8) | 0.8 (0.9) | *0.13* | 0.5 (0.7) | 0.2 (0.5) | *0.15* |
| Type of stent | 0.3 (0.6) | 0.4 (0.7) | *0.31* | 0.5 (0.6) | 0.1 (0.6) | *0.04* |

CA; coronary angiography, RoCA; rotational coronary angiography. Data presented as mean (SD).
