# Supplementary material for: When is rotational angiography superior to conventional single‐plane angiography for planning coronary angioplasty?
Source: Catheter Cardiovasc Interv. 2015 May 27;87(4):E104–12. doi: 10.1002/ccd.26032 (PMC4855622; doi:10.1002/ccd.26032)
Supplement: Supplementary file 3 — Supporting Information [file CCD-87-E104-s003.docx]

29th March 2015

Re-submission for *Catheterization and Cardiovascular Interventions*

**When is rotational angiography superior to conventional single plane angiography for planning coronary angioplasty?**

Paul D Morris^1,2,3^ BMedSci MBChB MRCP, Jane Taylor^1^ BMedSci MBChB, Sara Boutong^1^ BMedSci MBChB, Sarah Brett^2^ MD FRCP, Amal Louis^2^ MD MRCP, James Heppenstall^2^ DCR(R) PGCert, Allison C Morton^2^ PhD FRCP, Julian P Gunn^1,2,3^ MD MRCP

^1^Dept of Cardiovascular Science, University of Sheffield, UK. ^2^Dept of Cardiology, Sheffield Teaching Hospitals, Sheffield, UK. ^3^Insigneo Institute for *In Silico* Medicine, Sheffield, UK

Corresponding author: Dr Paul D Morris,

Medical Physics Group, Department of Cardiovascular Science, University of Sheffield, S10 2RX, [paul.morris@sheffield.ac.uk](mailto:paul.morris@sheffield.ac.uk)

Running title: Rotational angiography in PCI

Word count: 3257 (manuscript, including tables and figure legends)

Figures: 3

Tables: 4

E-appendix 1

Conflict of interest: The authors have no conflicts of interest to declare.

Key words: Coronary angiography, Rotational coronary angiography, Percutaneous coronary intervention

Abstract

**Objectives**

To investigate the value of rotational coronary angiography (RoCA) in the context of percutaneous coronary intervention (PCI) planning.

**Background**

As a diagnostic tool, RoCA is associated with decreased patient irradiation and contrast use compared with conventional coronary angiography (CA) and provides superior appreciation of 3-dimensional anatomy. However, its value in PCI remains unknown.

**Methods**

We studied stable CAD assessment and PCI planning by interventional cardiologists. Patients underwent either RoCA or conventional CA pre-PCI for planning. These were compared with the referral CA (all conventional) in terms of quantitative lesion assessment and operator confidence. An independent panel re-analysed all parameters.

**Results**

Six operators performed 127 procedures (60-RoCA; 60-conventional CA; 7-crossed-over) and assessed 212 lesions. RoCA was associated with a reduction in the number of lesions judged to involve a bifurcation (23 vs 30 lesions, p<0.05) and a reduction in the assessment of vessel caliber (2.8mm vs 3.0mm, p<0.05). RoCA improved confidence assessing lesion length (p=0.01), percentage stenosis (p=0.02), tortuosity (p<0.04) and proximity to a bifurcation (p=0.03), particularly in LCA cases. X-ray dose, contrast agent volume and procedure duration were not significantly different.

**Conclusions**

Compared with conventional CA, RoCA augments quantitative lesion assessment, enhances confidence in the assessment of coronary artery disease and the precise details of the proposed procedure, but does not affect X-ray dose, contrast agent volume or procedure duration.

A**bbreviations**

CA Coronary angiography

CAD Coronary artery disease

2-D 2-dimensional

3-D 3-dimensional

RoCA Rotational coronary angiography

PCI Percutaneous coronary intervention

LCA Left coronary artery

RCA Right coronary artery

**Introduction**

For over 50 years, invasive coronary angiography (CA) has been the gold standard investigation for diagnosing and assessing coronary artery disease (CAD). CA remains the only investigation capable of selecting patients for, and guiding, coronary revascularization. CA techniques and protocols continue to improve in terms of accessibility, temporo-spatial resolution, radiation dose reduction, image manipulation and data storage.

A limitation of conventional CA is that it consists of a series of 2-dimensional (2-D) ‘snapshots’ of the coronary arteries, acquired from a limited number of often restricted angles or 'planes'. Appreciating the true 3-dimensional (3-D) coronary and lesion anatomy requires the operator to recall the appearance of previously recorded angiographic runs and ‘reconstruct’ the 3-D anatomy using their imagination. This is subjective and can be unreliable (1-3). A number of studies based on intravascular ultrasound, angioscopy, and postmortem analysis have demonstrated how conventional, single-plane CA may fail to adequately represent various anatomical characteristics, particularly in the context of complex coronary artery disease (CAD) (4-9).

Biplane angiography offers a partial solution by recording from two orthogonal angiographic planes simultaneously. Although it is associated with reduced contrast use, the equipment required is not available in every center, the X-ray dose can be high, and the number of views obtainable can be limited. Consequently, this technique has become more a feature of non-coronary, structural heart intervention (10).

Rotational coronary angiography (RoCA) is a relatively new method of angiographic image acquisition originally conceived and developed for imaging cerebral vessels, to overcome the limitations of conventional angiography (11-13). During RoCA, images are acquired as the X-ray C-arm rotates around the patient, in a transverse axis, (typically an arc of 120^0^, at 30^0^ per second), recording 121 sequential 2-D images, with or without cranial or caudal tilt (Figure 1 and 2) (14). RoCA has several advantages over traditional CA. First, in the context of diagnostic CA, RoCA is associated with a reduction in the volume of contrast agent used and the total radiation dose (14-17). RoCA has therefore gained popularity in patients with renal insufficiency (18). Second, RoCA provides 121 separate views, and therefore may reveal more anatomical detail than conventional CA. Third, RoCA is viewed as a single run, whereas conventional CA requires the operator or radiographer to ‘scroll’ between multiple single plane acquisitions (19). Fourth, using image segmentation software, RoCA image data can be used to generate a 3-D reconstruction of vessel geometry which can be manipulated on a desktop computer to aid planning of percutaneous coronary intervention (PCI) (20). More recently, this technique has been exploited to generate 3-D geometric models for computing intra-coronary physiological parameters such as fractional flow reserve (FFR) (21).

Immediately prior to PCI, a ‘planning’ CA is performed which assesses any changes since the diagnostic CA, any unclear aspects of the coronary circulation or lesion and to determine the optimal strategy for PCI. RoCA has become established in the context of diagnostic CA, but its role in pre-PCI planning is yet to be established. The aim of the current study was to compare RoCA and conventional CA in the context of pre-PCI strategy planning in terms of lesion assessment, operator confidence, procedure time, volume of contrast agent infused and radiation dose delivered.

**Materials and Methods**

*Location and design*

This was an observational study performed at the South Yorkshire Cardiothoracic Centre, Sheffield Teaching Hospitals NHS Foundation Trust. The study complied with local research ethics committee guidance.

*Population*

Patients with stable, native vessel CAD referred for PCI were studied. Patients with graft lesions, chronic total occlusion, acute presentation or severe truncal obesity were excluded.

*Clinical protocol*

Patients were selected for standard CA or RoCA planning according to operator preference (non-random), maintaining a balance between both techniques. Operators were accustomed to both techniques. Baseline clinical data were extracted from hospital records. Referral CAs (all conventional) were evaluated, by the operator, who recorded details of the lesion, the proposed PCI strategy and their level of confidence (0-10, 10 indicating highest confidence) regarding each element of angiographic assessment and PCI planning. Operators re-evaluated these assessments, on the basis of the planning angiogram (RoCA or standard CA) before proceeding to PCI according to standard practice. X-ray dosage, contrast usage and procedure time were recorded. Operators also graded their level of confidence associated with each angiographic method in terms of lesion assessment and PCI planning. Planning CAs were then compared with referral CAs, to evaluate any added value associated with the different techniques in terms of planning PCI. All parameters were re-evaluated by an independent panel. Figure 1 outlines the study protocol. Details of the patient and PCI evaluation record are included in the e-appendix.

*Angiographic protocols*

Conventional multiple, single-plane CA was performed according to standard practice. Individual projection angles were selected at the operators’ discretion. RoCA was performed using the Philips system, after iso-centering in posterior-anterior and lateral planes on a breath hold, with a single hand injection of 15 to 20 ml contrast. Right coronary artery (RCA) target vessels underwent a single RoCA with 25° cranial tilt and left coronary artery (LCA) cases underwent two RoCAs, one with 25° cranial tilt and one with 25° caudal tilt, to ensure comprehensive assessment of all lesions and branches.

*Statistics*

Continuous data are expressed as mean (standard deviation) and were compared using paired or unpaired Student’s t-tests, as appropriate. Categorical data are expressed as number and/or percentage and were compared using Pearson’s chi squared test or the Wilcoxon sign-rank test. Between-group differences in confidence scores were compared using the Mann-Whitney U test. Fisher’s exact test was used to analyze 2 x 2 tables. Statistical significance was considered at the 5% level.

**Results**

Six PCI operators performed 127 procedures over a seven month period. 66 patients underwent RoCA and 61 conventional CA as their planning angiogram. All six operators contributed cases to both groups with a balance between conventional and rotational techniques (JG; 25&27, SB; 19&16, AL; 8&7, TR; 5&7, others; 4&9). Seven patients (5.5%; 6 RoCA & 1 conventional CA) crossed-over to the other modality, which could be regarded as ‘failure’ of the initial strategy as a complete assessment tool (p=0.11). These seven were excluded from subsequent analyses. The mean patient age was 64 years, weight; 80 kg, height; 1.70 m and 62% were male. The mean number of significant lesions was 1.8, vessels treated; 1.5, balloons used; 2.8, and stents deployed; 2.0. There were no significant differences between the two groups in terms of baseline demographics, medical history, or type of PCI procedure performed (Table 1). In total, 212 significant lesions were assessed for potential treatment (97 conventional, 115 RoCA; 61 RCA, 151 LCA).

*Lesion assessment and PCI strategy*

Some lesions which had previously been judged by the operators on the basis of the referral CA as non-significant were deemed, during the planning CA, to be significant, and vice versa. However, there was no significant difference in the frequency of lesions being excluded or additional lesions included between the RoCA and conventional groups (see appendix Table A1).

*Quantitative lesion assessment*

Paired comparison analysis of lesion characteristics (Table 2) revealed that using RoCA to plan PCI resulted in a significantly different assessment of vessel caliber and lesion involvement with a bifurcation, compared with the referral CA. Operators and the independent panel found that, compared with the conventional referral CA, using RoCA led to a reduction in the assessment of vessel caliber (2.8 mm vs 3.0 mm, p<0.05) and a reduction in the number of lesions deemed to involve a bifurcation (23 vs 30 lesions, p<0.05). Using conventional CA to plan PCI did not alter lesion characteristic assessment.

*Confidence levels*

Operator confidence in all aspects of the planning was significantly greater with RoCA compared with conventional CA (confidence level on a scale of 0-10 was 8.9 vs 8.2, respectively; p<0.05). In terms of the perceived number of significant lesions, both the operators and the independent expert panel were more confident following RoCA compared with conventional CA (increase in confidence level 0.9 vs 0.5 for the operators, 0.6 vs 0.3 for the panel; p<0.05 for both). In the assessment of certain lesion characteristics (lesion length, % stenosis, tortuosity and angulation) there was also a greater increase in confidence amongst operators and the panel with RoCA compared with conventional CA (Table 3).

*Left vs right coronary artery*

Amongst operators and the panel, there was a greater increase in confidence level in assessing lesion characteristics (both RoCA and conventional CA) for LCA cases, compared with the RCA cases. However, there was added value in terms of increased confidence in assessing several lesion characteristics (length, % stenosis, irregularity and angulation) with RoCA rather than conventional CA in the LCA cases (Table A1). This was not the case in RCA cases (Table A3).

*Procedure time, radiation dose and contrast volume*

There were no statistically significant differences in total procedure time, X-ray dose, screening time, cine runs or contrast usage between PCIs guided by the two techniques, although all trends favored conventional CA over RoCA (Table 4).

*Supplemental diagnostic runs*

31 out of 60 (52%) planning RoCAs were supplemented with an additional single plane (conventional) acquisition. Most commonly (22 cases), this was a supplementary left anterior oblique (LAO) caudal (‘spider’) view to better assess the distal left main stem and proximal left anterior descending artery and circumflex. In two out of 60 (3%) conventional CA cases, RoCA was added.

**Discussion**

This is the first study to investigate the use of RoCA in the context of planning PCI; comparing RoCA against conventional single-plane CA for immediate pre-PCI planning. RoCA improved the operator’s understanding of the target lesions and the proposed procedure compared with the knowledge gained from the diagnostic (conventional) CA. RoCA resulted in a reduction in the number of lesions judged to involve a bifurcation and a reduction in the assessed vessel caliber. These parameters directly influence PCI strategy, particularly in terms of stent sizing and deployment. RoCA may therefore provide additional information in terms of quantitative lesion measurements and proposed stent parameters. Whether or not this corresponds with a reduction in complications and negative outcomes (arising from stent over-sizing and procedures which unnecessarily involve a bifurcation) remains to be determined.

A further advantage of RoCA was in the level of confidence demonstrated by the operator in terms of understanding of the 3-D anatomy and the lesion characteristics, particularly in the LCA. The parameters in which this was noted were lesion length, percentage stenosis, lesion irregularity and the degree of tortuosity. Unlike in diagnostic CA, planning RoCA inferred no advantages in terms of reducing X-ray dose, contrast usage, nor procedure or screening time.

Any advantage conferred by a greater confidence level when performing PCI is unclear, and this study did not aim to address that. It may be speculated that improved confidence might translate into better patient safety or even long term results, although it would require much larger large study to prove that. There might also be an economic advantage, if the correct stent type and length is selected, or by deploying a more trackable device in a tortuous vessel. A particular advantage of RoCA vs conventional CA was observed in assessment of the LCA rather than the RCA, presumably because of greater 3-D complexity manifested in vessel overlap and branching in the LCA, which is not the case in the RCA. No advantage was found when RoCA, rather than conventional CA, was used in the RCA.

RoCA has been installed in many cardiac catheter laboratories, and is a well-established method of imaging the coronary arteries (16,22). Yet it is rarely used, either in the diagnostic role or in the role of PCI planning. The reasons are well known to operators who use the technique, and may be inferred from the trend observed in our study towards an increased procedure time with RoCA which occurs because of the requirement for careful iso-centering and a ‘dummy run’ and the frequent proximity warnings (activated if the C-arm approaches the patient) which necessitate adjustments to drapes, patient position and table height prior re-starting the whole process. In addition, errors in iso-centering (cutting off a vessel), insufficient contrast in the syringe, or disengagement of the catheter requires RoCA to be repeated. Even good quality RoCAs may have to be supplemented with conventional runs, (52%in the current study) usually because the degree of cranial or caudal angulation is insufficient to visualize the proximal LCA. Finally, RoCA is impossible in very obese patients.

There was no advantage in terms of radiation dose, contrast usage or procedure time with RoCA compared with conventional CA in the pre-PCI planning role, despite reductions in these parameters being documented in the role of diagnostic CA in several studies (14-17). The reasons for this are two-fold. First, regarding contrast and X-ray dose, the pre-PCI set-up CA is only one small part of the procedure; conventional single plane CAs are used to guide the procedure as it progresses in both groups. Therefore any small savings in contrast and radiation at the first stage will be diluted by the expenditure of each during the course of the procedure. Second, regarding time, accurate setting up of a good quality RoCA takes longer than acquiring 3 or 4 single plane conventional CAs.

*Limitations of this study*

This was an observational, rather than a randomized study. However, operators were competent at both conventional CA and RoCA and selected the modality themselves in approximately equal numbers without significant bias. An attempt to limit any potential bias was made by incorporating a parallel analysis by a panel, offline; and also the baseline characteristics of the patients, vessels and lesions in each group were remarkably similar. This was a single center study, although this conferred the advantage of consistency of methodology. The numbers included were modest. Outcome measures related to operator assessments rather than patient outcomes.

**Conclusions**

In the context of planning PCI, RoCA may offer advantages of better appreciation of lesions and planning of the procedure than conventional CA, particularly in left coronary artery cases. In contrast to purely diagnostic RoCA, it does not save reduce the volume of contrast used, the X-ray exposure or the procedure time. It has several practical disadvantages in a busy interventional catheter laboratory. It is not known whether these procedural advantages translate into clinical benefit for patients or training benefit for interventional cardiologists.

**Acknowledgments**

None

**Funding**

Dr Morris is supported by a British Heart Foundation Research Training Fellowship (R/134747-11-1). Dr Taylor received a research award from Heart Research UK (Registered Charity No.1044821).

**Conflict of interests**

None of the authors have any conflict of interest to declare.

**References**

1. Green NE, Chen SY, Messenger JC, Groves BM, Carroll JD. Three-dimensional vascular angiography. Curr Probl Cardiol 2004;29(3):104-42.

2. Fleming RM, Kirkeeide RL, Smalling RW, Gould KL. Patterns in visual interpretation of coronary arteriograms as detected by quantitative coronary arteriography. J Am Coll Cardiol 1991;18(4):945-51.

3. Bertrand ME, Lablanche JM, Bauters C, Leroy F, Mac Fadden E. Discordant results of visual and quantitative estimates of stenosis severity before and after coronary angioplasty. Cathet Cardiovasc Diagn 1993;28(1):1-6.

4. Schwartz JN, Kong Y, Hackel DB, Bartel AG. Comparison of angiographic and postmortem findings in patients with coronary artery disease. Am J Cardiol 1975;36(2):174-8.

5. Arnett EN, Isner JM, Redwood DR, Kent KM, Baker WP, Ackerstein H, Roberts WC. Coronary artery narrowing in coronary heart disease: comparison of cineangiographic and necropsy findings. Ann Intern Med 1979;91(3):350-6.

6. Isner JM, Kishel J, Kent KM, Ronan JA, Jr., Ross AM, Roberts WC. Accuracy of angiographic determination of left main coronary arterial narrowing. Angiographic--histologic correlative analysis in 28 patients. Circulation 1981;63(5):1056-64.

7. Mizuno K, Miyamoto A, Satomura K, Kurita A, Arai T, Sakurada M, Yanagida S, Nakamura H. Angioscopic coronary macromorphology in patients with acute coronary disorders. Lancet 1991;337(8745):809-12.

8. De Scheerder I, De Man F, Herregods MC, Wilczek K, Barrios L, Raymenants E, Desmet W, De Geest H, Piessens J. Intravascular ultrasound versus angiography for measurement of luminal diameters in normal and diseased coronary arteries. Am Heart J 1994;127(2):243-51.

9. Mintz GS, Painter JA, Pichard AD, Kent KM, Satler LF, Popma JJ, Chuang YC, Bucher TA, Sokolowicz LE, Leon MB. Atherosclerosis in angiographically "normal" coronary artery reference segments: an intravascular ultrasound study with clinical correlations. J Am Coll Cardiol 1995;25(7):1479-85.

10. Bashore TM, Balter S, Barac A, Byrne JG, Cavendish JJ, Chambers CE, Hermiller JB, Jr., Kinlay S, Landzberg JS, Laskey WK and others. 2012 American College of Cardiology Foundation/Society for Cardiovascular Angiography and Interventions expert consensus document on cardiac catheterization laboratory standards update: A report of the American College of Cardiology Foundation Task Force on Expert Consensus documents developed in collaboration with the Society of Thoracic Surgeons and Society for Vascular Medicine. J Am Coll Cardiol 2012;59(24):2221-305.

11. Cornelis G, Bellet A, van Eygen B, Roisin P, Libon E. Rotational multiple sequence roentgenography of intracranial aneurysms. Acta Radiol Diagn (Stockh) 1972;13(1):74-6.

12. Thron A, Voigt K. Rotational cerebral angiography: procedure and value. AJNR Am J Neuroradiol 1983;4(3):289-91.

13. Tu RK, Cohen WA, Maravilla KR, Bush WH, Patel NH, Eskridge J, Winn HR. Digital subtraction rotational angiography for aneurysms of the intracranial anterior circulation: injection method and optimization. AJNR Am J Neuroradiol 1996;17(6):1127-36.

14. Horisaki TK, O. Imai, S. Inada, T. Suzuki, T. Iinuma, K. Sugiura, H. Bakker, N. Melman, N. Feasibility evaluation of dual axis rotational angiography (XperSwing) in the diagnosis of coronary artery disease. MEDICAMUNDI 2008;52(2).

15. Raman SV, Morford R, Neff M, Attar TT, Kukielka G, Magorien RD, Bush CA. Rotational X-ray coronary angiography. Catheter Cardiovasc Interv 2004;63(2):201-7.

16. Maddux JT, Wink O, Messenger JC, Groves BM, Liao R, Strzelczyk J, Chen SY, Carroll JD. Randomized study of the safety and clinical utility of rotational angiography versus standard angiography in the diagnosis of coronary artery disease. Catheter Cardiovasc Interv 2004;62(2):167-74.

17. Rigattieri S, Ghini AS, Silvestri P, Tommasino A, Ferraiuolo G, Palamara A, Loschiavo P. A randomized comparison between rotational and standard coronary angiography. Minerva Cardioangiol 2005;53(1):1-6.

18. Kuon E, Niederst PN, Dahm JB. Usefulness of rotational spin for coronary angiography in patients with advanced renal insufficiency. Am J Cardiol 2002;90(4):369-73.

19. Tommasini G, Camerini A, Gatti A, Derchi G, Bruzzone A, Vecchio C. Panoramic coronary angiography. J Am Coll Cardiol 1998;31(4):871-7.

20. Liao R, Luc D, Sun Y, Kirchberg K. 3-D reconstruction of the coronary artery tree from multiple views of a rotational X-ray angiography. Int J Cardiovasc Imaging 2010;26(7):733-49.

21. Morris PD, Ryan D, Morton AC, Lycett R, Lawford PV, Hose DR, Gunn JP. Virtual Fractional Flow Reserve From Coronary Angiography: Modeling the Significance of Coronary Lesions: Results From the VIRTU-1 (VIRTUal Fractional Flow Reserve From Coronary Angiography) Study. JACC Cardiovasc Interv 2013;6(2):149-57.

22. Empen K, Kuon E, Hummel A, Gebauer C, Dorr M, Konemann R, Hoffmann W, Staudt A, Weitmann K, Reffelmann T and others. Comparison of rotational with conventional coronary angiography. Am Heart J 2010;160(3):552-63.

**Figure legends**

*Figure 1*

Representative frames from a typical rotational angiogram of a left coronary artery recorded in the caudal projection. The frame number is shown in each case.

*Figure 2*

Representative frames from a typical rotational angiogram of a right coronary artery recorded in the cranial projection. The frame number is shown in each case.

*Figure 3*

Flowchart of the study protocol (blue) and data collection (green).
